# Bis(2-carboxy­anilinium) sulfate monohydrate

**DOI:** 10.1107/S1600536810008913

**Published:** 2010-03-13

**Authors:** Taslim Akhtar, Khawar Masih, M. Nawaz Tahir, Muhammad Ilyas Tariq, Shahid Iqbal

**Affiliations:** a Department of Chemistry, University of Sargodha, Sargodha, Pakistan; b Department of Physics, University of Sargodha, Sargodha, Pakistan

## Abstract

In the title hydrated mol­ecular salt, 2C_7_H_8_NO_2_^+^·SO_4_^2−^·H_2_O, each cation in the asymmetric unit is stabilized by an intra­molecular N—H⋯O hydrogen bond. The O atoms of the sulfate ion are disordered over two sets of sites with an occupancy ratio of 0.541 (13):0.459 (13), which possibly optimizes the acceptance of N—H⋯O hydrogen bonds from the cations. The crystal structure also features aromatic π–π stacking [centroid–centroid separation = 3.842 (2) Å] and a C—H⋯π inter­action.

## Related literature

For background to the properties and uses of amino­benzoic acids, see: Griss *et al.* (1984 ▶); Pedanova *et al.* (1984 ▶); Refaat (2010 ▶); Rogers & Clark (1973 ▶).
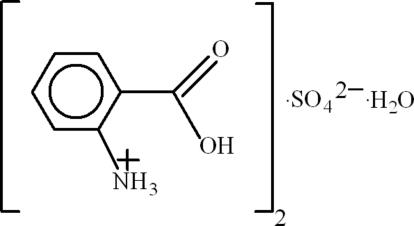


## Experimental

### 

#### Crystal data

2C_7_H_8_NO_2_^+^·SO_4_^2−^·H_2_O
*M* *_r_* = 390.36
Monoclinic, 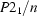

*a* = 11.260 (5) Å
*b* = 10.542 (4) Å
*c* = 15.358 (5) Å
β = 109.737 (5)°
*V* = 1715.9 (12) Å^3^
*Z* = 4
Mo *K*α radiation
μ = 0.24 mm^−1^
*T* = 296 K
0.28 × 0.25 × 0.20 mm

#### Data collection

Bruker Kappa APEXII CCD diffractometer
Absorption correction: multi-scan (*SADABS*; Bruker, 2005 ▶) *T*_min_ = 0.934, *T*_max_ = 0.955
12530 measured reflections
3748 independent reflections
2528 reflections with *I* > 2σ(*I*)
*R*_int_ = 0.036

#### Refinement

*R*[*F* ^2^ > 2σ(*F* ^2^)] = 0.046
*wR*(*F* ^2^) = 0.118
*S* = 1.02
3748 reflections
282 parameters
H atoms treated by a mixture of independent and constrained refinement
Δρ_max_ = 0.22 e Å^−3^
Δρ_min_ = −0.40 e Å^−3^

### 

Data collection: *APEX2* (Bruker, 2007 ▶); cell refinement: *SAINT* (Bruker, 2007 ▶); data reduction: *SAINT*; program(s) used to solve structure: *SHELXS97* (Sheldrick, 2008 ▶); program(s) used to refine structure: *SHELXL97* (Sheldrick, 2008 ▶); molecular graphics: *ORTEP-3 for Windows* (Farrugia, 1997 ▶) and *PLATON* (Spek, 2009 ▶); software used to prepare material for publication: *WinGX* (Farrugia, 1999 ▶) and *PLATON*.

## Supplementary Material

Crystal structure: contains datablocks global, I. DOI: 10.1107/S1600536810008913/hb5357sup1.cif

Structure factors: contains datablocks I. DOI: 10.1107/S1600536810008913/hb5357Isup2.hkl

Additional supplementary materials:  crystallographic information; 3D view; checkCIF report

## Figures and Tables

**Table 1:** Hydrogen-bond geometry (Å, °) *Cg*2 is the centroid of of the C8–C13 ring.

*D*—H⋯*A*	*D*—H	H⋯*A*	*D*⋯*A*	*D*—H⋯*A*
N1—H1*A*⋯O6*A*^i^	0.89	1.83	2.721 (6)	174
N1—H1*B*⋯O2	0.89	1.99	2.708 (3)	137
N1—H1*B*⋯O4^ii^	0.89	2.33	3.041 (3)	137
N1—H1*C*⋯O8*A*^iii^	0.89	1.98	2.860 (11)	168
N2—H2*A*⋯O8*A*^iv^	0.89	1.83	2.698 (12)	166
N2—H2*B*⋯O4	0.89	1.94	2.689 (3)	140
N2—H2*B*⋯O2^ii^	0.89	2.28	2.906 (3)	128
N2—H2*C*⋯O5*A*^i^	0.89	2.00	2.839 (6)	157
O1—H1⋯O9^v^	0.82	1.75	2.557 (3)	168
O3—H3*A*⋯O7*A*^v^	0.82	1.70	2.512 (13)	167
O9—H9*A*⋯O6*A*^vi^	0.80 (3)	2.46 (3)	3.102 (8)	138 (3)
O9—H9*A*⋯O8*A*^vi^	0.80 (3)	2.38 (4)	3.115 (11)	153 (3)
O9—H9*B*⋯O5*A*	0.86 (3)	1.96 (3)	2.792 (7)	161 (3)
C4—H4⋯*Cg*2^vii^	0.93	2.75	3.600 (3)	153

Symmetry codes: (i) 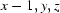; (ii) 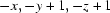; (iii) 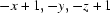; (iv) 
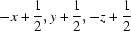
; (v) 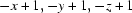; (vi) 
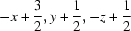
; (vii) 
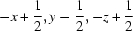
.

**Fig. 1.**
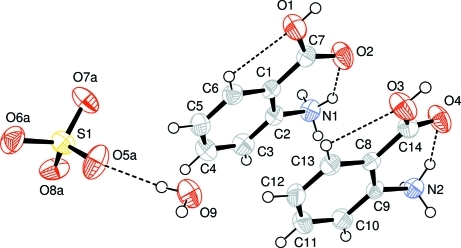
View of (I) with displacement ellipsoids drawn at the 50% probability level. The dotted lines show the hydogen bonds.

## supplementary crystallographic information

### Comment

The salts of aminobenzoic acids have been reported as medicines as well as precursor for in the field of pharmaceutics (Griss *et al.*, 1984). These are also useful for autoxidation of the fats forming free radicals, hence, in comparison to sulfated metal oxides, these can be used for production of biodiesel (Pedanova *et al.*, 1984; Refaat, 2010). The structure of title compound (I, Fig. 1) is being reported here.

In the title compound (I) benzene rings A (C1–C6) and B (C8—C13) are of course planar with maximum r. m. s. deviations of 0.0025 and 0.0034 Å from the mean squares planes. The dihedral angle between A/B is 7.91 (13)°. The carboxylate groups C (O1/C7/O2) and D (O3/C14/O4) are oriented at dihedral angles of 11.94 (38)° and 10.86 (41)° with benzene rings A and B respectively. The O-atoms of SO_4_^-2^ are disordered over two set of sites with occupancy ratio of 0.541 (13):0.459 (13). The molecules are stabilized due to intra as well as inter-molecular H-bondings and C–H···π interactions (Table 1, Fig. 2). The π–π interaction between the centroids CgA and CgB of benzene rings A and B respectively, also play a role in the stabilization of molecules. The centroid to centroid distance is 3.842 (2) Å.

### Experimental

Ethanolic solution of anthranilic acid (0.02 M) was refluxed in the presence of conc. H_2_SO_4_ (0.01 M) for 30 min. The contents were kept at room temperature for 24 h. The crystalline material formed was washed with n-hexane, ethyl acetate and diethyl ether, respectively and dried. This material was dissolverd in ethanol and colorless prisms of (I) were obtainesd by slow evaporation at room temperature.

### Refinement

Although all H-atoms were recognised from the difference fourier map but only coordinates of H-atoms of H_2_O were refined. The H-atoms were positioned geometrically (C—H = 0.93, O—H = 0.82, N—H = 0.89 Å) and refined as riding with *U*_iso_(H) = x*U*_eq_(C, N, O), where x = 1.5 for NH_3_ and hydroxy H atoms, whereas x = 1.2 for all other H-atoms.

### Crystal data

**Table:**

2C_7_H_8_NO_2_^+^·SO_4_^2^^−^·H_2_O	*F*(000) = 816
*M**_r_* = 390.36	*D*_x_ = 1.511 Mg m^−^^3^
Monoclinic, *P*2_1_/*n*	Mo *K*α radiation, λ = 0.71073 Å
Hall symbol: -P 2yn	Cell parameters from 3752 reflections
*a* = 11.260 (5) Å	θ = 2.0–27.1°
*b* = 10.542 (4) Å	µ = 0.24 mm^−^^1^
*c* = 15.358 (5) Å	*T* = 296 K
β = 109.737 (5)°	Prisms, colorless
*V* = 1715.9 (12) Å^3^	0.28 × 0.25 × 0.20 mm
*Z* = 4	

### Data collection

**Table:**

Bruker Kappa APEXII CCD diffractometer	3748 independent reflections
Radiation source: fine-focus sealed tube	2528 reflections with *I* > 2σ(*I*)
graphite	*R*_int_ = 0.036
Detector resolution: 7.50 pixels mm^-1^	θ_max_ = 27.1°, θ_min_ = 2.0°
ω scans	*h* = −14→14
Absorption correction: multi-scan (*SADABS*; Bruker, 2005)	*k* = −12→13
*T*_min_ = 0.934, *T*_max_ = 0.955	*l* = −19→18
12530 measured reflections	

### Refinement

**Table:**

Refinement on *F*^2^	Primary atom site location: structure-invariant direct methods
Least-squares matrix: full	Secondary atom site location: difference Fourier map
*R*[*F*^2^ > 2σ(*F*^2^)] = 0.046	Hydrogen site location: inferred from neighbouring sites
*wR*(*F*^2^) = 0.118	H atoms treated by a mixture of independent and constrained refinement
*S* = 1.02	*w* = 1/[σ^2^(*F*_o_^2^) + (0.0551*P*)^2^ + 0.3168*P*] where *P* = (*F*_o_^2^ + 2*F*_c_^2^)/3
3748 reflections	(Δ/σ)_max_ < 0.001
282 parameters	Δρ_max_ = 0.22 e Å^−^^3^
0 restraints	Δρ_min_ = −0.40 e Å^−^^3^

### Special details

**Table:**

Geometry. Bond distances, angles etc. have been calculated using the rounded fractional coordinates. All su's are estimated from the variances of the (full) variance-covariance matrix. The cell esds are taken into account in the estimation of distances, angles and torsion angles
Refinement. Refinement of *F*^2^ against ALL reflections. The weighted *R*-factor *wR* and goodness of fit *S* are based on *F*^2^, conventional *R*-factors *R* are based on *F*, with *F* set to zero for negative *F*^2^. The threshold expression of *F*^2^ > σ(*F*^2^) is used only for calculating *R*-factors(gt) *etc*. and is not relevant to the choice of reflections for refinement. *R*-factors based on *F*^2^ are statistically about twice as large as those based on *F*, and *R*- factors based on ALL data will be even larger.

### Fractional atomic coordinates and isotropic or equivalent isotropic displacement parameters (Å^2^)

**Table:**

	*x*	*y*	*z*	*U*_iso_*/*U*_eq_	Occ. (<1)
O1	0.45161 (14)	0.45846 (15)	0.61664 (12)	0.0529 (6)	
O2	0.24999 (14)	0.41658 (17)	0.58921 (13)	0.0613 (7)	
N1	0.15921 (15)	0.19068 (17)	0.51077 (12)	0.0408 (6)	
C1	0.36494 (17)	0.28683 (19)	0.51932 (13)	0.0326 (6)	
C2	0.27346 (18)	0.19261 (19)	0.48532 (14)	0.0346 (6)	
C3	0.2899 (2)	0.0970 (2)	0.43001 (16)	0.0459 (8)	
C4	0.3988 (2)	0.0928 (2)	0.40722 (16)	0.0518 (8)	
C5	0.4909 (2)	0.1840 (2)	0.44025 (15)	0.0478 (8)	
C6	0.47351 (18)	0.2800 (2)	0.49500 (14)	0.0404 (7)	
C7	0.34884 (18)	0.3926 (2)	0.57799 (14)	0.0357 (6)	
O3	0.20126 (14)	0.69772 (16)	0.43653 (12)	0.0569 (6)	
O4	0.01902 (14)	0.62640 (15)	0.44221 (12)	0.0557 (6)	
N2	−0.09794 (14)	0.43896 (16)	0.32614 (12)	0.0364 (6)	
C8	0.12219 (17)	0.51165 (19)	0.35454 (13)	0.0307 (6)	
C9	0.02378 (18)	0.42708 (18)	0.31295 (13)	0.0308 (6)	
C10	0.0387 (2)	0.3301 (2)	0.25830 (15)	0.0412 (7)	
C11	0.1524 (2)	0.3134 (2)	0.24375 (16)	0.0488 (8)	
C12	0.2513 (2)	0.3945 (2)	0.28460 (15)	0.0442 (7)	
C13	0.23563 (18)	0.4927 (2)	0.33872 (14)	0.0376 (6)	
C14	0.10804 (19)	0.6174 (2)	0.41481 (14)	0.0367 (7)	
S1	0.82551 (5)	0.10652 (5)	0.37522 (4)	0.0404 (2)	
O5A	0.7633 (7)	0.2145 (4)	0.3274 (4)	0.0608 (16)	0.541 (13)
O6A	0.9595 (4)	0.1055 (9)	0.3666 (3)	0.0576 (19)	0.541 (13)
O7A	0.8109 (9)	0.1137 (12)	0.4669 (9)	0.053 (2)	0.541 (13)
O8A	0.7732 (9)	−0.0123 (10)	0.3402 (8)	0.051 (2)	0.541 (13)
O5B	0.6994 (8)	0.1600 (10)	0.3072 (4)	0.078 (3)	0.459 (13)
O6B	0.9227 (7)	0.1737 (8)	0.3650 (3)	0.0500 (19)	0.459 (13)
O7B	0.8590 (9)	0.1102 (15)	0.4772 (10)	0.049 (2)	0.459 (13)
O8B	0.8163 (12)	−0.0309 (13)	0.3482 (10)	0.057 (3)	0.459 (13)
O9	0.55554 (17)	0.37630 (18)	0.26338 (13)	0.0535 (7)	
H1	0.43834	0.51227	0.65081	0.0793*	
H1A	0.09356	0.16783	0.46196	0.0612*	
H1B	0.14569	0.26764	0.52934	0.0612*	
H1C	0.16899	0.13539	0.55646	0.0612*	
H3	0.22814	0.03516	0.40785	0.0551*	
H4	0.40991	0.02812	0.36949	0.0622*	
H5	0.56438	0.18049	0.42545	0.0573*	
H6	0.53543	0.34189	0.51640	0.0484*	
H2A	−0.15481	0.46829	0.27447	0.0546*	
H2B	−0.09109	0.49252	0.37237	0.0546*	
H2C	−0.12228	0.36332	0.33962	0.0546*	
H3A	0.18855	0.75376	0.46936	0.0854*	
H10	−0.02800	0.27507	0.23078	0.0495*	
H11	0.16195	0.24753	0.20641	0.0586*	
H12	0.32830	0.38297	0.27570	0.0531*	
H13	0.30250	0.54790	0.36542	0.0451*	
H9A	0.576 (3)	0.407 (3)	0.2229 (19)	0.0642*	
H9B	0.607 (2)	0.313 (3)	0.2786 (18)	0.0642*	

### Atomic displacement parameters (Å^2^)

**Table:**

	*U*^11^	*U*^22^	*U*^33^	*U*^12^	*U*^13^	*U*^23^
O1	0.0400 (8)	0.0520 (11)	0.0720 (12)	−0.0148 (8)	0.0259 (8)	−0.0236 (9)
O2	0.0370 (9)	0.0661 (12)	0.0872 (13)	−0.0092 (8)	0.0294 (9)	−0.0292 (10)
N1	0.0334 (9)	0.0396 (11)	0.0503 (11)	−0.0066 (8)	0.0153 (8)	0.0028 (9)
C1	0.0296 (10)	0.0350 (12)	0.0325 (10)	−0.0007 (9)	0.0097 (8)	0.0052 (9)
C2	0.0318 (10)	0.0353 (12)	0.0369 (11)	0.0001 (9)	0.0117 (9)	0.0064 (10)
C3	0.0463 (12)	0.0388 (13)	0.0513 (14)	−0.0073 (11)	0.0147 (11)	−0.0023 (11)
C4	0.0571 (15)	0.0498 (15)	0.0506 (14)	0.0039 (12)	0.0209 (12)	−0.0097 (12)
C5	0.0390 (12)	0.0606 (16)	0.0478 (13)	0.0031 (12)	0.0200 (10)	−0.0015 (12)
C6	0.0316 (10)	0.0486 (14)	0.0401 (11)	−0.0039 (10)	0.0111 (9)	−0.0012 (11)
C7	0.0306 (10)	0.0365 (12)	0.0403 (11)	−0.0015 (9)	0.0125 (9)	0.0048 (10)
O3	0.0507 (9)	0.0519 (10)	0.0768 (13)	−0.0231 (8)	0.0328 (9)	−0.0311 (9)
O4	0.0425 (9)	0.0538 (10)	0.0792 (12)	−0.0122 (8)	0.0314 (9)	−0.0287 (9)
N2	0.0299 (9)	0.0365 (10)	0.0410 (10)	−0.0058 (8)	0.0095 (8)	−0.0005 (8)
C8	0.0290 (9)	0.0297 (11)	0.0302 (10)	−0.0005 (9)	0.0059 (8)	0.0015 (9)
C9	0.0304 (10)	0.0279 (11)	0.0315 (10)	0.0023 (9)	0.0070 (8)	0.0038 (9)
C10	0.0428 (12)	0.0328 (12)	0.0439 (12)	−0.0038 (10)	0.0091 (10)	−0.0067 (10)
C11	0.0585 (15)	0.0395 (13)	0.0495 (14)	0.0089 (12)	0.0196 (12)	−0.0070 (11)
C12	0.0386 (12)	0.0509 (14)	0.0453 (12)	0.0107 (11)	0.0169 (10)	0.0033 (12)
C13	0.0302 (10)	0.0409 (12)	0.0389 (11)	−0.0025 (9)	0.0082 (9)	0.0007 (10)
C14	0.0322 (10)	0.0341 (12)	0.0425 (12)	−0.0052 (9)	0.0111 (9)	−0.0047 (10)
S1	0.0493 (3)	0.0288 (3)	0.0455 (3)	−0.0044 (3)	0.0192 (3)	−0.0041 (3)
O5A	0.045 (3)	0.033 (2)	0.096 (3)	0.004 (2)	0.013 (2)	0.020 (2)
O6A	0.0306 (19)	0.080 (5)	0.061 (2)	−0.002 (2)	0.0141 (16)	0.007 (2)
O7A	0.075 (5)	0.039 (3)	0.058 (4)	−0.011 (5)	0.041 (5)	−0.014 (3)
O8A	0.065 (4)	0.026 (4)	0.048 (2)	−0.013 (3)	−0.001 (3)	−0.006 (2)
O5B	0.046 (4)	0.084 (5)	0.093 (4)	0.018 (4)	0.009 (3)	0.019 (3)
O6B	0.045 (3)	0.046 (4)	0.064 (3)	−0.015 (3)	0.025 (2)	0.005 (2)
O7B	0.061 (5)	0.043 (3)	0.047 (3)	−0.006 (5)	0.025 (5)	−0.009 (2)
O8B	0.099 (8)	0.028 (3)	0.057 (6)	−0.003 (5)	0.045 (6)	−0.004 (3)
O9	0.0570 (11)	0.0506 (11)	0.0635 (12)	−0.0020 (8)	0.0343 (9)	−0.0038 (9)

### Geometric parameters (Å, °)

**Table:**

S1—O7A	1.474 (13)	N2—H2B	0.8900
S1—O8A	1.411 (11)	C1—C2	1.399 (3)
S1—O5B	1.557 (8)	C1—C7	1.483 (3)
S1—O6B	1.356 (8)	C1—C6	1.396 (3)
S1—O7B	1.483 (14)	C2—C3	1.370 (3)
S1—O5A	1.407 (5)	C3—C4	1.385 (3)
S1—O6A	1.559 (5)	C4—C5	1.379 (3)
S1—O8B	1.501 (14)	C5—C6	1.371 (3)
O1—C7	1.308 (3)	C3—H3	0.9300
O2—C7	1.209 (3)	C4—H4	0.9300
O1—H1	0.8200	C5—H5	0.9300
O3—C14	1.301 (3)	C6—H6	0.9300
O4—C14	1.215 (3)	C8—C13	1.393 (3)
O3—H3A	0.8200	C8—C14	1.492 (3)
O9—H9A	0.80 (3)	C8—C9	1.397 (3)
O9—H9B	0.86 (3)	C9—C10	1.369 (3)
N1—C2	1.466 (3)	C10—C11	1.383 (3)
N1—H1A	0.8900	C11—C12	1.376 (3)
N1—H1C	0.8900	C12—C13	1.376 (3)
N1—H1B	0.8900	C10—H10	0.9300
N2—C9	1.457 (3)	C11—H11	0.9300
N2—H2A	0.8900	C12—H12	0.9300
N2—H2C	0.8900	C13—H13	0.9300
			
O5B—S1—O7B	123.3 (6)	C4—C5—C6	119.7 (2)
O5B—S1—O8B	101.6 (7)	C1—C6—C5	121.4 (2)
O6B—S1—O7B	100.4 (5)	O1—C7—C1	113.84 (18)
O6B—S1—O8B	117.1 (6)	O1—C7—O2	122.7 (2)
O7B—S1—O8B	106.7 (8)	O2—C7—C1	123.5 (2)
O5A—S1—O8A	116.7 (5)	C4—C3—H3	120.00
O6A—S1—O7A	120.3 (5)	C2—C3—H3	120.00
O6A—S1—O8A	104.7 (6)	C3—C4—H4	120.00
O7A—S1—O8A	104.1 (7)	C5—C4—H4	120.00
O5B—S1—O6B	108.8 (5)	C4—C5—H5	120.00
O5A—S1—O6A	106.7 (4)	C6—C5—H5	120.00
O5A—S1—O7A	105.1 (6)	C5—C6—H6	119.00
C7—O1—H1	109.00	C1—C6—H6	119.00
C14—O3—H3A	109.00	C9—C8—C13	117.46 (18)
H9A—O9—H9B	100 (3)	C9—C8—C14	121.75 (18)
C2—N1—H1B	109.00	C13—C8—C14	120.79 (18)
H1B—N1—H1C	109.00	N2—C9—C10	117.75 (18)
H1A—N1—H1B	109.00	N2—C9—C8	121.31 (17)
C2—N1—H1A	109.00	C8—C9—C10	121.0 (2)
H1A—N1—H1C	109.00	C9—C10—C11	120.4 (2)
C2—N1—H1C	109.00	C10—C11—C12	119.9 (2)
C9—N2—H2C	109.00	C11—C12—C13	119.6 (2)
C9—N2—H2B	109.00	C8—C13—C12	121.7 (2)
C9—N2—H2A	109.00	O4—C14—C8	123.2 (2)
H2A—N2—H2C	109.00	O3—C14—C8	113.54 (19)
H2A—N2—H2B	109.00	O3—C14—O4	123.3 (2)
H2B—N2—H2C	109.00	C11—C10—H10	120.00
C2—C1—C7	122.35 (18)	C9—C10—H10	120.00
C2—C1—C6	117.69 (18)	C10—C11—H11	120.00
C6—C1—C7	119.96 (18)	C12—C11—H11	120.00
N1—C2—C3	118.01 (19)	C13—C12—H12	120.00
N1—C2—C1	120.82 (18)	C11—C12—H12	120.00
C1—C2—C3	121.1 (2)	C12—C13—H13	119.00
C2—C3—C4	119.8 (2)	C8—C13—H13	119.00
C3—C4—C5	120.3 (2)		
			
C6—C1—C2—N1	177.63 (18)	C13—C8—C9—N2	179.56 (18)
C6—C1—C2—C3	0.0 (3)	C13—C8—C9—C10	−0.5 (3)
C7—C1—C2—N1	−3.4 (3)	C14—C8—C9—N2	0.4 (3)
C7—C1—C2—C3	179.0 (2)	C14—C8—C9—C10	−179.60 (19)
C2—C1—C6—C5	−0.5 (3)	C9—C8—C13—C12	−0.2 (3)
C7—C1—C6—C5	−179.48 (19)	C14—C8—C13—C12	178.92 (19)
C2—C1—C7—O1	168.59 (19)	C9—C8—C14—O3	−170.35 (18)
C2—C1—C7—O2	−11.6 (3)	C9—C8—C14—O4	10.8 (3)
C6—C1—C7—O1	−12.4 (3)	C13—C8—C14—O3	10.5 (3)
C6—C1—C7—O2	167.4 (2)	C13—C8—C14—O4	−168.3 (2)
N1—C2—C3—C4	−177.61 (19)	N2—C9—C10—C11	−179.52 (19)
C1—C2—C3—C4	0.1 (3)	C8—C9—C10—C11	0.5 (3)
C2—C3—C4—C5	0.3 (3)	C9—C10—C11—C12	0.2 (3)
C3—C4—C5—C6	−0.8 (3)	C10—C11—C12—C13	−0.8 (3)
C4—C5—C6—C1	0.9 (3)	C11—C12—C13—C8	0.9 (3)

### Hydrogen-bond geometry (Å, °)

**Table:**

Cg2 is the centroid of of the C8–C13 ring.

**Table:**

*D*—H···*A*	*D*—H	H···*A*	*D*···*A*	*D*—H···*A*
N1—H1A···O6A^i^	0.89	1.83	2.721 (6)	174
N1—H1B···O2	0.89	1.99	2.708 (3)	137
N1—H1B···O4^ii^	0.89	2.33	3.041 (3)	137
N1—H1C···O8A^iii^	0.89	1.98	2.860 (11)	168
N2—H2A···O8A^iv^	0.89	1.83	2.698 (12)	166
N2—H2B···O4	0.89	1.94	2.689 (3)	140
N2—H2B···O2^ii^	0.89	2.28	2.906 (3)	128
N2—H2C···O5A^i^	0.89	2.00	2.839 (6)	157
O1—H1···O9^v^	0.82	1.75	2.557 (3)	168
O3—H3A···O7A^v^	0.82	1.70	2.512 (13)	167
O9—H9A···O6A^vi^	0.80 (3)	2.46 (3)	3.102 (8)	138 (3)
O9—H9A···O8A^vi^	0.80 (3)	2.38 (4)	3.115 (11)	153 (3)
O9—H9B···O5A	0.86 (3)	1.96 (3)	2.792 (7)	161 (3)
C4—H4···Cg2^vii^	0.93	2.75	3.600 (3)	153

Symmetry codes: (i) *x*−1, *y*, *z*; (ii) −*x*, −*y*+1, −*z*+1; (iii) −*x*+1, −*y*, −*z*+1; (iv) −*x*+1/2, *y*+1/2, −*z*+1/2; (v) −*x*+1, −*y*+1, −*z*+1; (vi) −*x*+3/2, *y*+1/2, −*z*+1/2; (vii) −*x*+1/2, *y*−1/2, −*z*+1/2.
